# Association of GERD with Sleeve Gastrectomy: An Unintended Consequence

**DOI:** 10.1007/s11892-025-01617-y

**Published:** 2026-01-29

**Authors:** Jonanne Talebloo, Kishore M. Gadde, Ravinder K. Mittal, Ninh T. Nguyen

**Affiliations:** 1 Department of Surgery, Division of Gastrointestinal Surgery, University of California Irvine School of Medicine, 3800 W Chapman Ave, Suite 6200, Orange, CA 92868 USA; 2 Department of Medicine, Division of Gastroenterology and Hepatology, University of California San Diego School of Medicine, 9500 Gilman Dr, La Jolla, CA 92093 USA

**Keywords:** Obesity, Bariatric surgery, Sleeve gastrectomy, Gastroesophageal reflux disease, GERD

## Abstract

**Purpose of Review:**

Bariatric surgery is a highly effective treatment for obesity that yields durable weight loss with significant improvement or resolution of T2D and other weight-related chronic cardiometabolic diseases. While the advantages of laparoscopic sleeve gastrectomy (LSG), the most performed bariatric surgery procedure, include procedural simplicity, short operating time, lower complication rate, durable weight loss, and significant improvement including remission of type 2 diabetes, a major drawback is gastroesophageal reflux disease (GERD). The purpose of this review is to summarize the prevalence of and predictors of GERD after LSG, physiological mechanisms that explain the risk, and novel surgical management and strategy.

**Recent Findings:**

Studies note high rates of *de novo* GERD and worsening of pre-existing GERD following LSG; however, estimates vary due to inconsistent definitions and length of follow-ups across the cohorts. Physiological studies demonstrate that LSG increases intragastric pressure and esophageal acid exposure in conjunction with specific anatomic alterations, which together can explain the rise in reflux seen postoperatively. Preoperative reflux, including undiagnosed preoperative GERD, is the strongest predictor of postoperative GERD. For patients with persistent GERD symptoms, conversion to gastric bypass is a common treatment, and experimental work suggests that adaptations of principles from fundoplication to sleeve anatomy can offer a pathway to minimize LSG-induced reflux.

**Summary:**

Future studies should be aimed at determining which elements of the antireflux barrier that must be preserved or reconstructed to reduce reflux after LSG. Additionally, there is a need to fully understand how the mechanics of fundoplication can be adapted and applied to sleeve anatomy to create a reliable antireflux barrier.

## Introduction

Obesity prevalence continues to rise in the United States and globally. During August 2021 - August 2023, the estimated prevalence of obesity among US adults was 40.3% with 9.4% meeting the threshold for severe obesity [1]. Globally, in 2021, an estimated 1.0 billion adult males and 1.1 billion adult females had overweight or obesity [2]. Obesity is associated with a heavy burden of chronic diseases, including type 2 diabetes (T2D), hypertension, dyslipidemia, cardiovascular disease, metabolic dysfunction-associated steatotic liver disease (MASLD), and obstructive sleep apnea [3]. Of most concern is the alarming increase in global prevalence of diabetes. Globally, 1.31 billion people are projected to have diabetes, with 96% of the cases being T2D, which is tightly linked to excess body weight [4].

### Obesity, Diabetes, and Gastroesophageal Reflux Disease (GERD)

At the population level, GERD is the most common gastrointestinal disease with an estimated prevalence of 18–28% in the US [5, 6]. Other data suggest the burden may be considerably higher, with a 2020 nationwide Cedars-Sinai survey of over 71,000 adults finding that 44.1% of adults reported experiencing GERD symptoms at some point [7]. GERD is even more common among patients with obesity, with reported prevalence rates as high as 70% [8, 9]. One meta-analysis confirmed that obesity significantly increases the risk of GERD and estimated that patients with a BMI of ≥30 kg/m^2^ have a 1.94 times higher risk for GERD compared with those with a BMI of < 25 kg/m^2^ [10]. Several mechanisms have been proposed to explain the relationship between obesity and GERD, including defective lower esophageal sphincter (LES), abnormal esophageal clearance, increased intra-abdominal pressure, and higher prevalence of hiatal hernia [11]. Chronic GERD increases the risk of Barrett’s esophagus, which may progress to esophageal adenocarcinoma (EAC) [12, 13]. A pooled analysis of 12 epidemiological studies found an almost 5-fold increase (odds ratio [OR] 4.8, 95% CI 3.0–7.7.0.7) in the risk for EAC in individuals with BMI ≥40 kg/m^2^ compared to those with BMI < 25 kg/m^2^ [14]. The risk of GERD is significantly increased in patients with diabetes with one meta-analysis reporting an OR of 1.61 (1.36–1.91) [15]. Esophageal gastric dysfunction and delayed gastric emptying have been suggested as possible mechanisms of increased reflux [16]. Although weight loss is expected to decrease gastroesophageal reflux associated with obesity and diabetes, glucagon-like peptide-1 receptor agonists, which induce significant weight loss, are often associated with worsening of GERD [17].

### Bariatric Surgery is Effective

Bariatric surgery remains the most effective treatment for obesity and has been demonstrated in numerous long-term studies and systematic reviews to result in durable weight loss with significant improvement or resolution of T2D, hypertension, dyslipidemia, MASLD, and reduce major adverse cardiovascular events [18–26]. Bariatric surgery is currently included in the treatment algorithm for T2D [27]. Treatments that integrate surgery, lifestyle modification and pharmacotherapy are often utilized in long-term weight management.

Over 600,000 bariatric surgeries were performed annually worldwide and approximately 270,000 in the US in 2023, with laparoscopic sleeve gastrectomy (LSG) accounting for 58% and Roux-en-Y gastric bypass (RYGB) 23%, with revisional surgeries and other laparoscopic and endoscopic procedures accounting for the remaining 19% [28, 29].

### Association of LSG and GERD

LSG fails to improve GERD and may, in many cases, worsen pre-existing reflux or trigger *de novo* GERD even among patients who achieved significant weight loss [30–33]. In a nine-year follow-up study, data showed that reflux symptoms worsened over time following LSG [34]. Medium-term endoscopic data from a five-year gastroscopy cohort show that reflux prevalence rose from 16% to 64% at five years post-LSG, including 54% of those cases reporting de novo GERD [35]. Earlier literature assessing GERD after LSG was limited by small single-center cohorts, heterogeneous operative techniques, variable diagnostic definitions, and inclusion of heterogeneous studies in systematic reviews [33, 36–39]. More recently, large observational studies and randomized clinical trials have provided clearer insights into the prevalence of GERD following LSG. A large nationwide register-based cohort study in Denmark with an average follow-up of 4 years found that 37% of patients commenced proton-pump inhibitor (PPI) therapy after LSG [40]. Long-term data from randomized clinical trials first emerged in 2018 with the publication of the SM-BOSS and SLEEVEPASS trials, both of which demonstrated increased incidence of reflux and esophagitis after LSG compared with RYGB [41, 42].

### Predictors of GERD after LSG

The most important predictor of GERD after LSG is undiagnosed preoperative GERD. A pH-monitoring study found that among patients with latent GERD before LSG, 76% developed GERD symptoms [43]. In a study of more than 500,000 sleeve gastrectomy patients (2015–2019), preoperative reflux predicted short-term morbidity and long-term reflux vulnerability [44]. Findings from a cohort of patients who completed preoperative Bravo wireless esophageal pH testing suggest that elevated markers of acid exposure, rather than standard definitions of abnormal reflux, may serve as more appropriate thresholds for identifying patients at higher risk of GERD after LSG [42]. In this same study, higher preoperative acid exposure predicted postoperative reflux, suggesting that threshold-based stratification with routine preoperative pH testing may be warranted in patients planning to undergo LSG [45]. A 2024 nomogram study in 236 patients found that a model that combined diabetes, hiatal hernia, serum triglyceride level, BMI, and pre-existing GERD predicted worsening GERD after LSG [46]. Physiologic testing has also been shown to predict risks. A study combining high-resolution manometry with preoperative pH monitoring showed that patients with reduced LES pressure and weaker esophageal motility were prone to postoperative GERD [47]. In a single-center cohort of 213 patients, higher severity of preoperative heartburn predicted postoperative GERD, while a somewhat protective factor was higher preoperative BMI [48]. A prospective endoscopy-based evaluation of 217 patients undergoing sleeve gastrectomy showed that mild preoperative esophagitis and small hiatal hernia significantly increase the likelihood of postoperative GERD [49]. Taken together, these findings show that a deliberate, detailed preoperative workup can be central to identifying patients with increased risk of GERD after LSG and to help guide alternative bariatric surgical procedures, and incorporate appropriate follow-up in the treatment plan.

### Anatomy and Physiology of the Normal Antireflux Barrier (ARB)

**Fig. 1:**
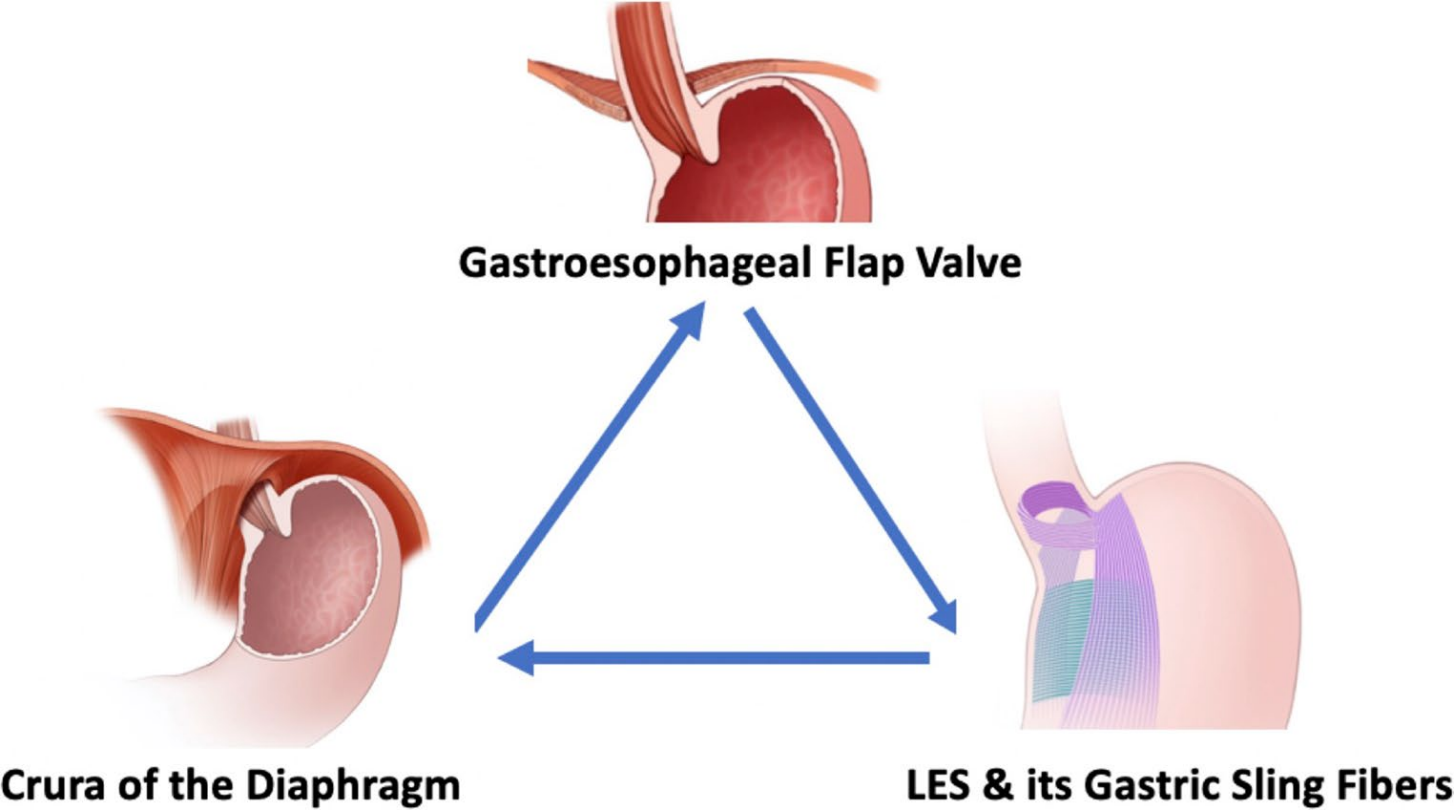
The three components of the antireflux barrier (ARB)

There are three important components of a normal ARB (Fig. 1): the smooth muscle lower esophageal sphincter [LES], the skeletal muscle of the crural diaphragm (CD), and the gastroesophageal flap valve (GEFV) [50–53].

The right crura muscle that originates from the lumbar spine (L1-L3) divides into two bundles to form the opening in the diaphragm (esophageal hiatus) that allows the esophagus to enter from the chest into the abdomen. The LES and CD are anatomically superimposed and are anchored to each other by the phrenoesophageal ligament. The latter originates from the lower and upper surfaces of the diaphragm. Attenuation of the phrenoesophageal ligament leads to cephalad migration of the LES into the chest (sliding hiatus hernia), which in turn leads to the loss of the acute angle of His. The LES and CD provide circumferential closure mechanisms at the esophagogastric junction (EGJ), which is related to their unique myoarchitecture. The circular muscle of the distal esophagus crosses at the angle of His and continues into the stomach as the sling fibers of the LES. The smooth muscle LES generates tonic contraction that is related to its unique myogenic properties. In addition, neural elements, excitatory and inhibitory, modulate the LES tone. The CD contraction during the inspiratory phase of the respiratory cycle and physical maneuvers that increase abdominal pressure (abdominal compression, straight leg raise, Valsalva maneuver) increase the LES pressure. The LES or EGJ pressure (as measured by manometry) is a measure of the strength of the ARB; the higher the EGJ pressure, the greater the strength of the ARB. An individual would need higher gastric pressure than EGJ pressure to promote gastroesophageal reflux (GER). The GEFV is part of the fundus of the stomach that rises above the EGJ and abuts against the distal esophagus. It allows gastric pressure to be exerted upon the distal esophagus. It is thought to be an important component of the ARB. Physiological studies show that transient LES relaxation is an important mechanism of GER. A low LES tone and impaired CD contraction are also important in the pathophysiology of GERD. Disruption of the esophagogastric morphology, i.e., development of sliding hiatal hernia, loss of intraabdominal esophageal length, exposure of LES to negative intrathoracic pressure, and disruption/loss of the GEFV are important elements of an incompetent ARB [54].

### Effects of LSG on the Esophagus

Whereas the ARB is often impaired in patients with obesity, LSG causes additional iatrogenic disruption of the esophagogastric morphology. Specifically, LSG alters the anatomy of the angle of His, eliminates the natural GEFV, potentially divides the gastric sling fibers, and creates a smaller-diameter gastric tube that increases the intragastric pressure [55, 56]. LSG surgically divides the gastric cardia at the angle of His, thus leading to the loss of the GEFV, which is a critical component of the ARB. It also potentially disrupts the gastric sling fibers of the LES. In the context that bariatric surgery patients are often found to have an impaired ARB to begin with, further disruption of the ARB components by the LSG may explain the increased GERD risk with this surgery. In summary, the mechanism for the development of pathologic reflux after LSG is likely related to anatomic disruption of the gastric sling fibers, which can impair the LES function, and division of the stomach at the angle of His, leading to the loss of the naturally-occurring GEFV. Lastly, impairment of these two barriers in a patient with existing hiatal disruption likely contributes to worsening of existing GERD or development of *de novo* GERD.

New data from high-resolution esophageal manometry and pH impedance monitoring now offer a more detailed understanding of esophageal function and reflux burden after LSG [57, 58]. The 24-hour impedance and pH monitoring show a significant increase in reflux from 47.2% before LSG to 88.7% after surgery [57]. Physiological measures suggest that increases in gastric pressure, greater esophageal acid exposure, and decreased LES pressure are the mechanistic pathways underlying the increased GERD risk after LSG [57]. In a randomized clinical trial comparing LSG to one-anastomosis gastric bypass (OAGB) surgery, employing high resolution impedance manometry (HRiM; Manoscan) and impedance pH monitoring (MII-pH; Sleuth multi-channel intraluminal impedance system), it was discovered that the average acid exposure time (AET) increased by 2.8% points (4.3% at baseline to 7.1% at Month 12) in the LSG group and decreased (3.3% at baseline to 1.5% at Month 12) in the OAGB group, i.e., a net between-group difference of 4.6% [58]. Furthermore, the LES tone decreased from 18.0 mmHg to 13.3 mmHg following LSG. Of note, patients with a preoperative diagnosis of GERD were excluded from this study.

### Surgical Techniques and Procedural Alternatives

Several operative strategies have been developed to mitigate reflux risk after sleeve gastrectomy rates [59]; they involve preserving or reconstructing elements of the ARB. An example is the endoscopic sleeve gastroplasty (ESG) procedure, which reduces the gastric volume but does not alter the angle of His, and preserves the naturally-occurring GEFV [60]. Another example is combining LSG with angle of His reconstruction (LSG-His). One study reported that the prevalence of GERD symptoms was 14% among patients who had LSG-His compared to 46% among those who underwent standard LSG [61]. Angle of His reconstruction restores the acute EGJ angle by suturing the gastric fundus to the left diaphragmatic crus, which restores the GEFV [61].

**Fig. 2:**
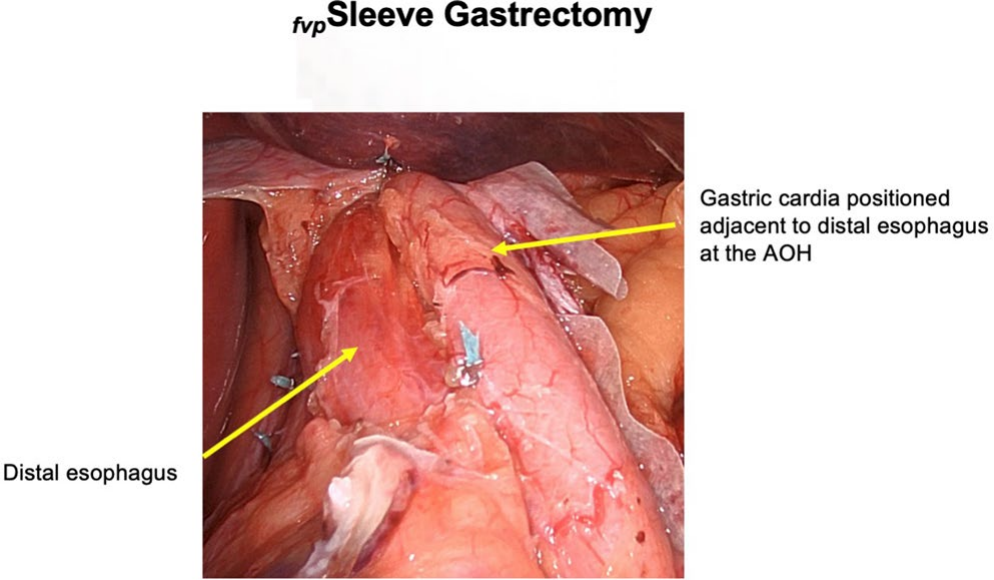
Completed Flap Valve-Preserving (fvp) Sleeve Gastrectomy: The preserved gastric cardia is sutured to the distal esophagus to reestablish an acute angle of His (AOH)

An important technical aspect of the LSG is to avoid narrowing of the gastric tube specifically at the junction where the gastric tube makes an anatomic bend [59]. Specifically, this technique avoids narrowing of the gastric tube at the gastric incisura, which is a known pressure bottleneck that increases intragastric pressure and induces reflux [62]. Finally, the concept of a flap-valve-preserving sleeve (*fvp*LSG, Fig. 2) was designed to increase the intraabdominal esophageal length, maintain the gastroesophageal complex during the sleeve construction, and restore the angle of His to preserve the GEFV. The INNOVATE-VSG pilot study is underway and aims to compare the effects of *fvp*LSG vs. conventional LSG on physiological and symptomatic measures of GERD [63].

In patients with refractory GERD following LSG, conversion to RYGB has been shown to improve reflux [64]. Nissen fundoplication is an effective antireflux surgery that emphasizes restoring adequate abdominal esophageal length, repairing the hiatus, reestablishing the angle of His, and augmenting the GEFV [65]. In a study of 58 patients who underwent Nissen fundoplication at the Veterans Administration hospitals by surgeons who followed 10 important principles of the Nissen, relief of symptoms occurred in 93% of patients and healing of esophagitis occurred in 77% cases [65]. In an ex-vivo study using a swine esophagus/stomach model, Lee et al. found that gastric distention following Nissen fundoplication (360-degree wrap) and Toupet fundoplication (270-degree wrap) increased the EGJ pressure and prevented reflux of gastric contents (gas or fluid) into the esophagus [66]. It was suggested that an increase in the diameter of the gastric fundus with gastric distension results in a constriction of the distal esophagus by the wrap. The magnitude of pressure increase at the EGJ during gastric distension can be explained based on Laplace’s law.

Emerging surgical approaches, including the combination of LSG with various types of conventional fundoplication (i.e., Nissen, Toupet, and Dor), require further evaluation because the data suggest increased complication rates, specifically increased risk for gastric perforation and suboptimal weight loss [67]. The risk for gastric perforation is related to gastric ischemia of the retained gastric fundus.

### Knowledge Gaps and Future Directions

Despite progress in understanding how sleeve gastrectomy is associated with GERD, there are considerable gaps in knowledge that must be addressed. A major unanswered question is which component of the ARB is most essential to preserve when modifying gastric anatomy. Future studies should focus on which of these factors most directly drive postoperative reflux and which elements of the ARB must be preserved or reconstructed to reduce the incidence of GERD.

A second major gap concerns how the mechanical principles of fundoplication can be adapted to sleeve anatomy. Experimental studies demonstrate that fundoplication creates a pressure-responsive barrier, in which gastric distension leads to a progressive rise in the EGJ pressure and a decrease in junction distensibility, preventing reflux even under high intragastric pressure [66]. An ongoing clinical trial aims to modify sleeve anatomy to preserve the GEFV to restore the elements of the ARB that are eliminated in the conventional sleeve LSG [63].

## Conclusions

Although LSG is the most widely performed bariatric surgery, a large proportion of patients develop new or worsened GERD postoperatively. This increase in reflux appears to be due to several anatomic and physiologic alterations created by sleeve gastrectomy, including creation of a smaller diameter gastric tube that elevates intragastric pressure, disruption of the angle of His, and concomitant loss of GEFV. Patients with preoperative reflux, particularly undiagnosed reflux, are at the highest risk for persistent GERD symptoms after LSG. Postoperative reflux is of clinical significance because it reduces quality of life, may lead to esophagitis and Barrett’s esophagus, and may increase the risk of EAC.

Traditional antireflux operations such as Nissen and Toupet fundoplication demonstrate how reconstruction of the angle of His can effectively prevent reflux in response to gastric distention, thus improving our understanding of the mechanism of antireflux surgery. Several operative strategies have been developed to address postoperative GERD by reconstructing or preserving components of the ARB. For example, endoscopic sleeve gastroplasty, hiatal hernia repair in conjunction with sleeve gastrectomy, sleeve construction that avoids narrowing of the gastric incisura, and the flap valve-preserving sleeve have shown promise in lowering postoperative reflux. For patients with significant postoperative reflux after LSG, conversion to RYGB is an option.

There is a need for rigorous prospective studies identifying which elements of the ARB are most important in promoting reflux control after LSG, as well as standardized preoperative physiologic testing to better identify patients at risk for postoperative reflux. Future work should also focus on developing and balancing innovative techniques to integrate important principles of fundoplication into sleeve gastrectomy to provide a stronger ARB while maintaining the principles of bariatric surgery to achieve optimal weight loss.

## Key References

- Sjöström L, Peltonen M, Jacobson P, Ahlin S, Andersson-Assarsson J, Anveden Å et al. Association of bariatric surgery with long-term remission of type 2 diabetes and with microvascular and macrovascular complications. JAMA. 2014;311(22):2297-304. 10.1001/jama.2014.5988.
  - ○ This prospective matched cohort study conducted in Sweden showed that patients who had bariatric surgery were 6 times more likely to achieve diabetes remission compared to control patients at 15-year follow-up.
- Peterli R, Wölnerhanssen BK, Peters T, Vetter D, Kröll D, Borbély Y et al. Effect of Laparoscopic Sleeve Gastrectomy vs Laparoscopic Roux-en-Y Gastric Bypass on Weight Loss in Patients With Morbid Obesity: The SM-BOSS Randomized Clinical Trial. JAMA. 2018;319(3):255-65. 10.1001/jama.2017.20897.
  - ○ This randomized trial found that Gastric reflux worsened more often after LSG (31.8%) than after RYGB (6.3%).
- Mittal RK, Kumar D, Kligerman SJ, Zifan A. Three-Dimensional Pressure Profile of the Lower Esophageal Sphincter and Crural Diaphragm in Patients with Achalasia Esophagus. Gastroenterology. 2020;159(3):864-72.e1. 10.1053/j.gastro.2020.05.017.
  - ○ This study provided detailed insights into the anatomy and physiology of the LES and the ARB.
- Nguyen NT, Gadde KM, Mittal RK. Flap Valve-Preserving Vertical Sleeve Gastrectomy (INNOVATE-VSG): Clinical Trial Study Protocol. Obes Surg. 2025;35(3):1063-9. 10.1007/s11695-025-07675-1.
  - ○ This paper describes the design of a clinical trial aimed at testing whether a flap valve-preserving LSG, compared to conventional LSG, can reduce GERD in patients with obesity and pre-existing GERD.

## Data Availability

No datasets were generated or analysed during the current study.
